# Smooth Muscle Hypocontractility and Airway Normoresponsiveness in a Mouse Model of Pulmonary Allergic Inflammation

**DOI:** 10.3389/fphys.2021.698019

**Published:** 2021-06-29

**Authors:** Magali Boucher, Cyndi Henry, Alexis Dufour-Mailhot, Fatemeh Khadangi, Ynuk Bossé

**Affiliations:** Institut Universitaire de Cardiologie et de Pneumologie de Québec – Université Laval, Québec, QC, Canada

**Keywords:** respiratory mechanics, airway responsiveness, mouse models, asthma, resistance

## Abstract

The contractility of airway smooth muscle (ASM) is labile. Although this feature can greatly modulate the degree of airway responsiveness *in vivo*, the extent by which ASM’s contractility is affected by pulmonary allergic inflammation has never been compared between strains of mice exhibiting a different susceptibility to develop airway hyperresponsiveness (AHR). Herein, female C57BL/6 and BALB/c mice were treated intranasally with either saline or house dust mite (HDM) once daily for 10 consecutive days to induce pulmonary allergic inflammation. The doses of HDM were twice greater in the less susceptible C57BL/6 strain. All outcomes, including ASM contractility, were measured 24 h after the last HDM exposure. As expected, while BALB/c mice exposed to HDM became hyperresponsive to a nebulized challenge with methacholine *in vivo*, C57BL/6 mice remained normoresponsive. The lack of AHR in C57BL/6 mice occurred despite exhibiting more than twice as much inflammation than BALB/c mice in bronchoalveolar lavages, as well as similar degrees of inflammatory cell infiltrates within the lung tissue, goblet cell hyperplasia and thickening of the epithelium. There was no enlargement of ASM caused by HDM exposure in either strain. Unexpectedly, however, excised tracheas derived from C57BL/6 mice exposed to HDM demonstrated a decreased contractility in response to both methacholine and potassium chloride, while tracheas from BALB/c mice remained normocontractile following HDM exposure. These results suggest that the lack of AHR in C57BL/6 mice, at least in an acute model of HDM-induced pulmonary allergic inflammation, is due to an acquired ASM hypocontractility.

## Introduction

The degree of airway responsiveness to a direct challenge, such as methacholine, is highly variable between individuals (Sparrow et al., 1987; Woolcock et al., 1987; Rijcken et al., 1988; Kennedy et al., 1990; Bakke et al., 1991; Paoletti et al., 1995; Ulrik, 1996). To tease out the underlying mechanisms liable for such disparity, different strains of mice exhibiting variable degrees of responsiveness have been widely utilized. While some strains are innately more responsive (Table 1), others rather display a different susceptibility to develop airway hyperresponsiveness (AHR) after exposure to offending triggers (Table 2). These murine models have been instrumental to deepen our understanding of the numerous elements contributing to AHR in diseases.

**TABLE 1:** Innate degree of airway responsiveness.

References	Mouse strains	Readouts
Levitt and Mitzner, 1988	A/J and C3H/HeJ	APTI
Levitt and Mitzner, 1989	A/J and C3H/HeJ	APTI
Gavett and Wills-Karp, 1993	A/J and C3H/HeJ	APTI
De Sanctis et al., 1995	A/J and C57BL/6	R_L_
Ewart et al., 1995	A/J and C3H/HeJ	Rrs, Ers, and APTI
Longphre and Kleeberger, 1995	AKR/J and C3H/HeJ	APTI
Ewart et al., 1996	A/J and C3H/HeJ	Rrs and APTI
De Sanctis et al., 1997	C57BL/6 and A/J	R_L_
Nicolaides et al., 1997	C57BL/6 and DBA/2	APTI
De Sanctis et al., 1999	A/J and C3H/HeJ	R_L_
Duguet et al., 2000	C57BL/6, BALB/c, A/J and C3H/HeJ	Rrs and Ers
Ackerman et al., 2005	C57BL/6 and A/J	Penh
Leme et al., 2010	36 strains, including C57BL/6 and BALB/c	R_N_
Berndt et al., 2011	29 strains, including C57BL/6 and BALB/c	Penh, R_N_, and H

*APTI, airway pressure time index; Ers, respiratory system elastance; H, tissue elastance; Penh, enhanced pause measured by whole-body plethysmography; R_*L*_, lung resistance; R_*N*_, Newtonian resistance; Rrs, respiratory system resistance.*

**TABLE 2:** Changes in airway responsiveness induced by offending triggers.

References	Mouse strains	Offending triggers	Readouts
Longphre and Kleeberger, 1995	AKR/J and C3H/HeJ	PAF	APTI
Zhang et al., 1997	C57BL/6 and BALB/c	OVA	G_L_ and C_L_
Miyabara et al., 1998	BALB/c and C3H/HeJ	DEP	R_*rs*_ and C_*rs*_
Brewer et al., 1999	12 strains, including C57BL/6 and BALB/c	OVA	G_L_
Zhang et al., 1999	BALB/c and BP2	OVA	Penh
Ewart et al., 2000	C57BL/6, BALB/c, A/J, AKR/J, and C3H/HeJ	OVA	APTI
McIntire et al., 2001	BALB/c and DBA/2	OVA	Penh
Takeda et al., 2001	C57BL/6 and BALB/c	OVA	R_L_ and C_L_
Kenyon et al., 2003	C57BL/6 and BALB/c	OVA	Penh
Shinagawa and Kojima, 2003	C57BL/6, BALB/c, A/J, and C3H/HeJ	OVA	Penh
Whitehead et al., 2003	9 strains, including C57BL/6 and BALB/c	OVA	Penh
Adler et al., 2004	C57BL/6 and BALB/c	OVA	Penh, R_L_, and C_L_
Koya et al., 2006	C57BL/6 and BALB/c	OVA	R_L_ and C_L_
Gueders et al., 2009	C57BL/6 and BALB/c	OVA	Penh and R_N_ (or Rrs – not clear)
Hirota et al., 2009	C57BL/6 and BALB/c	OVA	Rrs
Kearley et al., 2009	C57BL/6 and BALB/c	OVA	R_L_
Van Hove et al., 2009	C57BL/6 and BALB/c	OVA	Penh
Zhu and Gilmour, 2009	C57BL/6, BALB/c, and FVB/NJ	OVA	Penh
De Vooght et al., 2010	7 strains, including C57BL/6 and BALB/c	TDI	Rrs
Kodama et al., 2010	C57BL/6 and BALB/c	OVA	Rrs
Sahu et al., 2010	C57BL/6 and BALB/c	HDM	Rrs
Kelada et al., 2011	C57BL/6 and BALB/c	Der p 1	Rrs
Chang et al., 2013	C57BL/6 and BALB/c	Der f 2	Penh
Evans et al., 2015	C57BL/6 and BALB/c	OVA and Aspergillus	Rrs, R_N_, G and H
Li et al., 2017	C57BL/6 and BALB/c	FA and OVA	R_*i*_ and R_*e*_

*APTI, airway pressure time index; C_*L*_, lung compliance; Crs, respiratory system compliance; DEP, diesel exhaust particles; G, tissue damping; Ers, respiratory system elastance; FA, formaldehyde; G_*L*_, lung conductance; H, tissue elastance; OVA, ovalbumin; PAF, platelet-activating factor; Penh, enhanced pause measured by whole-body plethysmography; R_*e*_, expiratory resistance; R_*i*_, inspiratory resistance; R_*L*_, lung resistance; R_*N*_, Newtonian resistance; Rrs, respiratory system resistance; TDI, toluene-2,4-diisocyanate.*

One element that is mandatory for airway responsiveness is the contraction of airway smooth muscle (ASM). On the one hand, the contractility of ASM has been compared between strains of mice exhibiting different innate degrees of responsiveness. A/J mice, for example, have been shown to be hyperresponsive because of enhanced muscarinic signaling (Gavett and Wills-Karp, 1993) and excessive ASM shortening (Duguet et al., 2000; Wagers et al., 2007). On the other hand, the contractility of ASM has never been compared between mouse strains in studies showing a different susceptibility to develop AHR after exposure to offending triggers (Table 2). This is rather strange. We think it is due to the false perception that assessing airway responsiveness *in vivo* is a good surrogate for measuring ASM contractility. In fact, the level of ASM contractility rarely matches the degree of *in vivo* responsiveness in both mice (Weinmann et al., 1990) and humans (Armour et al., 1984a,b; Taylor et al., 1985; Cerrina et al., 1986; Roberts et al., 1987; Thomson, 1987; de Jongste et al., 1988; Whicker et al., 1988). ASM contractility should thus be regarded as one of many elements affecting the degree of *in vivo* airway responsiveness (Bosse et al., 2010).

It is also important to understand that the contractility of ASM is labile (Auger et al., 2016). The concept of lability stipulates that the contractility of ASM is not fixed but can rather change in response to different interventions. This lability is independent from a change in muscle size. It truly refers to an ASM of a given size being able to generate a force of different magnitudes in response to a given contractile stimulus. Typical examples of lability include the increased contractility caused by numerous inflammatory mediators (Auger et al., 2016).

The contractile lability of ASM may obviously contribute to the development of AHR observed in murine models of pulmonary allergic inflammation. The extent by which it occurs may also contribute to the different inter-strain susceptibility to develop AHR in a context of inflammation. Herein, we hypothesized that while ASM from BALB/c mice, a strain generally considered vulnerable for the development of AHR (Table 2), acquires hypercontractility in response to pulmonary allergic inflammation, the ASM from C57BL/6 mice, a strain generally considered less susceptible for the development of AHR (Table 2), remains normocontractile.

## Materials and Methods

### Mice

Sixty pathogen-free female C57BL/6 (Jackson, Bar Harbor, MA, United States) and 60 female BALB/c (Charles River, Saint-Constant, PQ, Canada) mice were purchased at 6- or 7-week-old. We chose females because they are more susceptible than males to the development of pulmonary allergic inflammation (Melgert et al., 2005; Blacquiere et al., 2010). They were provided food and water *ad libitum* and were housed until they reached 8 weeks of age before starting the protocol. All procedures were approved by the Committee of Animal Care of *Université Laval* in accordance with the guidelines of the Canadian Council on Animal Care (protocol 2018-046-2).

### Experimental Protocol

**FIGURE 1:**
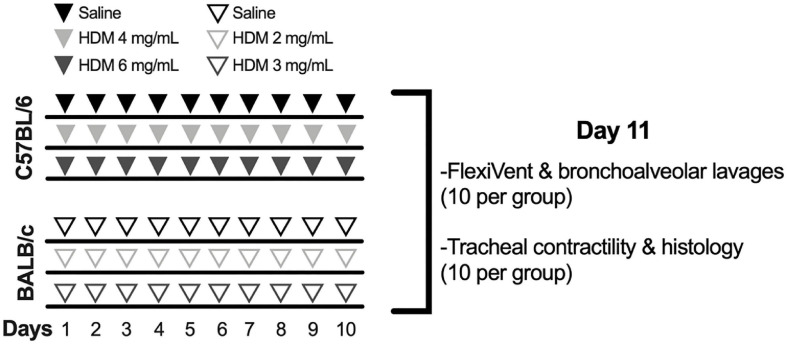
Experimental protocol to induce pulmonary allergic inflammation. While 60 C57BL/6 mice were exposed to saline (*n* = 20) or either 4 (*n* = 20) or 6 mg/mL (*n* = 20) of house dust mite (HDM), 60 BALB/c mice were exposed to saline (*n* = 20) or either 2 (*n* = 20) or 3 mg/mL (*n* = 20) of HDM once daily for 10 consecutive days. At day 11, the degree of *in vivo* airway responsiveness to methacholine was measured in half of the mice within each group (*n* = 10) using the flexiVent. The same mice were used to collect the bronchoalveolar lavages in order to measure cellular inflammation. The trachea was collected in the other half of mice within each group (*n* = 10), also at day 11, in order to measure the contractile capacity of airway smooth muscle in response to incremental concentrations of methacholine and potassium chloride. The left lung of the same mice was collected and processed for histology to quantify the infiltration of inflammatory cells within the tissue, the content of airway smooth muscle, the number of goblet cells and the thickness of the epithelium.

Mice from each strain were divided into three groups of 20 mice (Figure 1): one control group exposed to saline and two experimental groups exposed to one of two doses of house dust mite (HDM) extract (*Dermatophagoides pteronyssinus*; Greer, Lenoir, NC, United States). The endotoxin concentration was 47.3 EU per mg of HDM extract. They were exposed by intranasal instillation once daily for 10 consecutive days under isoflurane anesthesia. While C57BL/6 mice received 25 μL of 0, 4, or 6 mg of HDM extract per mL, BALB/c mice received 25 μL of 0, 2, or 3 mg of HDM extract per mL. HDM concentrations were twice higher in C57BL/6 mice in an attempt to aggravate inflammation and to promote AHR in this less susceptible strain (Table 2). All outcomes were measured 24 h after the last exposure. Half of the mice in each group (*n* = 10) was used to assess respiratory mechanics and to collect bronchoalveolar lavages (BAL). The other half was used to assess tracheal contractility and to collect the lung for histology (Figure 1).

### Respiratory Mechanics

Mice were anesthetized with ketamine (100 mg/Kg) and xylazine (10 mg/Kg). They were then tracheotomized and connected to the flexiVent (SCIREQ, Montreal, PQ, Canada) through an 18-gauge cannula in a supine position. To avoid leakage, a surgical thread was passed around the trachea and tightened to securely seal the tracheal wall against the cannula. The mice were also paralyzed by injecting pancuronium bromide (0.1 mg/Kg) intramuscularly. This was to avoid spontaneous breathing during the procedure. They were ventilated mechanically at a tidal volume of 10 mL/Kg, at a breathing frequency of 150 breaths/min and at a positive end expiratory pressure of 3 cmH_2_O.

Respiratory mechanics was assessed using the SnapShot-150 and the Quick Prime-3, two perturbation maneuvers inflicted by the flexiVent. The volume perturbation imparted by the SnapShot-150 is a single sine wave oscillation that allows one to infer values for resistance (Rrs) and elastance (Ers) of the respiratory system based on the linear single-compartment model (Bates, 2009). The volume perturbation imparted by the Quick Prime-3 is a composite signal, made of 13 sine waves of mutually prime frequencies and of different amplitudes and phases, that allows one to infer values for Newtonian resistance (R_N_), tissue damping (G), and tissue elastance (H) based on the constant phase model (Hantos et al., 1992).

The degree of airway responsiveness was assessed by nebulizing incremental concentrations of methacholine over 25 s at 5 min intervals. The concentrations used were 0, 3, 10, 30, and 100 mg/mL for C57BL/6 mice and 0, 1, 3, 10, and 30 mg/mL for BALB/c mice. These concentrations were tailored for each mouse strain according to their respective degree of airway responsiveness and to avoid doses that may be causing death (Mailhot-Larouche et al., 2018). Again, respiratory mechanics was assessed with the SnapShot-150 and the Quick Prime-3. Each of these volume-perturbation maneuvers was actuated 10 times in an alternating fashion after each dose, starting 10 s after dose delivery. Eight seconds of tidal breathing was intercalated between each volume-perturbation maneuver to avoid desaturation. A deep inflation was also imposed after the last volume-perturbation maneuver, ∼2 min before the subsequent dose. The peak value for each parameter (Rrs, Ers, R_N_, G, and H) after each dose were used to assess the response.

### Bronchoalveolar Lavage (BAL)

One mL of phosphate-buffered saline (PBS) was injected in the lung through the trachea and aspirated to recover the BAL. This was repeated three times and the recovered BAL was pooled together. The total volume was recorded and centrifuged at 500 × *g* for 5 min. The supernatant was discarded and the pellet was resuspended in 100 μL of PBS for control groups and 500 μL for HDM groups. Total cells in BAL were stained with crystal violet and counted using a hemacytometer. Seventy five thousand cells were also cytospin and stained with modified May-Grünwald Giemsa to count the number of macrophages, lymphocytes, neutrophils, and eosinophils.

### Tracheal Contractility

The whole trachea was collected and immersed in Krebs solution (pH 7.4, 111.9 mM NaCl, 5.0 mM KCl, 1.0 mm KH_2_PO_4_, 2.1 mM MgSO_4_, 29.8 mM NaHCO_3_, 11.5 mM glucose, and 2.9 mM CaCl_2_). It was then mounted horizontally in a 10-mL organ bath containing Krebs solution maintained at 37°C and coupled to a force transducer (Harvard Apparatus, St-Laurent, PQ, Canada). The latter measured the isometric force generated by the trachea in response to contractile activation. A resting distending force of 5 mN was applied. Before the contractile assays, the trachea was subjected to a period of conditioning, during which time it was stimulated to contract repeatedly for 5 min at 10-min intervals with 10^–5^ M of methacholine until a reproducible force was recorded.

Cumulative concentration-response curves were generated with methacholine and potassium chloride (KCl). Methacholine was added in log increments at 5-min intervals from 10^–7^ to 10^–4^ M. The concentration of KCl was doubled at 5-min intervals from 20 to 160 mM. The peak force obtained at each concentration was used to generate the concentration-response curves. The trachea was left untreated and washed repeatedly with fresh Krebs for at least 30 min between methacholine and KCl.

### Lung Histology

The left lung was excised and immersed in 4% paraformaldhehyde (PFA) during 24 h for fixation. The PFA was replaced by progressively upraising the ethanol concentration to dehydrate the tissue. The lung was then embedded in paraffin and cut transversally in 5 μm-thick sections. The sections were deposited on microscopic slides and stained with hematoxylin and eosin (H&E), Masson trichrome or Periodic acid-Schiff (PAS) with alcian blue. They were then scanned with a NanoZoomer Digital scanner (Hamamastu photonics, Bridgewater, NJ, United States) at 40X.

H&E stain was performed to evaluate the infiltration of inflammatory cells within the lung tissue. Fifteen non-overlapping photomicrographs (1440 × 904 pixels) from three non-contiguous lung sections were blindly scored from zero (no inflammation) to five (very severe inflammation) by one observer. The scores from each of the 15 photomicrographs were averaged to obtain one value per mouse and the values from each mouse within one group were compiled to obtain a mean per group.

Masson trichrome was used to quantify the content of smooth muscle within the airway wall. All bronchi cut transversally in three non-contiguous lung sections were analyzed, representing 1–6 bronchi per mouse (3.6 ± 1.5 and 3.3 ± 1.2 for C57BL/6 and BALB/c mice, respectively). The content of ASM in each bronchus was calculated by the area occupied by ASM divided by the square of the basement membrane perimeter. A mean was calculated for each mouse and the values from each mouse within one group were compiled to obtain a mean per group.

Periodic acid-Schiff with alcian blue was used to assess the number of goblet cells and the epithelium thickness. All bronchi cut transversally in three non-contiguous lung sections were analyzed, representing 1–7 bronchi per mouse (4.0 ± 1.9 and 2.6 ± 1.5 bronchi for C57BL/6 and BALB/c mice, respectively). The number of goblet cells within each bronchus was divided by its basement membrane perimeter. The epithelium thickness was analyzed by measuring the area occupied by the epithelium divided by the basement membrane perimeter. For both outcomes, a mean was calculated for each mouse and the values from each mouse within one group were compiled to obtain a mean per group.

### Data Analysis

Unless otherwise indicated, data are presented as means ± standard deviations. The parameters of respiratory mechanics and the readouts used for the contractile assays with excised tracheas were analyzed by repeated measures two-way ANOVA followed by Sidak’s multiple comparison tests. One-way ANOVA were used to compare inflammatory cells in BAL, the infiltration of inflammatory cells within the tissue, the content of ASM, counts of goblet cells and the epithelium thickness between groups. All statistical analyses were performed using Prism 9 (version 9.0.0, GraphPad, San Diego, CA, United States). *P* ≤ 0.05 was considered sufficient to reject the null hypothesis.

## Results

**FIGURE 2:**
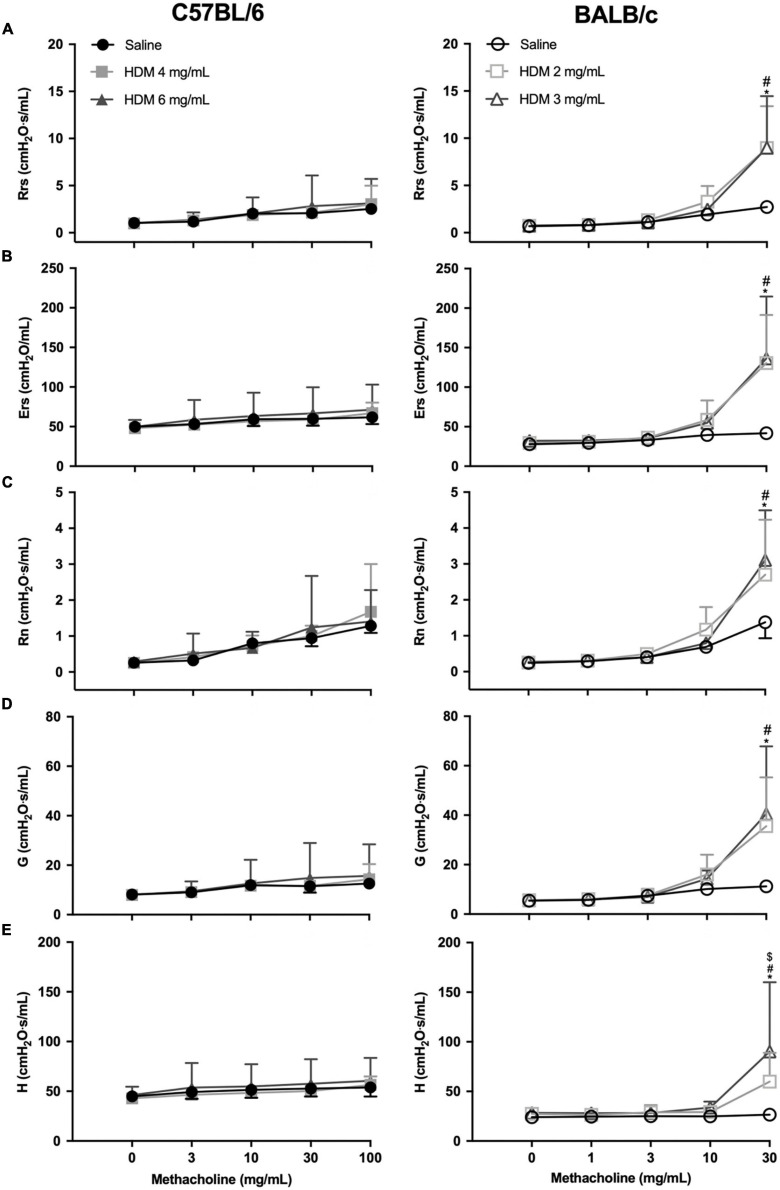
The degree of *in vivo* airway responsiveness in C57BL/6 (left panels) and BALB/c (right panels) mice exposed to either saline or one of two doses of HDM. Phosphate-buffered saline and incremental doses of methacholine were delivered by nebulization and the changes in several parameters were used to evaluate the degree of airway responsiveness, including: **(A)** respiratory system resistance (Rrs); **(B)** respiratory system elastance (Ers); **(C)** Newtonian resistance (R_N_); **(D)** tissue damping (G); and **(E)** tissue elastance (H). *, #, and $ designate significant differences in BALB/c mice for saline *vs.* HDM 3 mg/mL, for saline *vs.* HDM 2 mg/mL, and for HDM 3 *vs.* 2 mg/mL, respectively (*P* < 0.05). Data are shown as means ± SD (some error bars seem absent because their length is smaller than the symbol). *n* = 10 mice per group.

The degree of airway responsiveness to methacholine in C57BL/6 and BALB/c mice exposed to either saline or one of two doses of HDM is depicted in Figure 2. While BALB/c mice exposed to HDM developed AHR, C57BL/6 mice remained normoresponsive. This was true for all parameters used to assess respiratory mechanics, including Rrs, Ers, R_N_, G, and H. There was no significant difference in the degree of AHR between doses of 2 *vs.* 3 mg/mL of HDM in BALB/c mice, except for H, which reached a significantly higher value after the highest dose of methacholine for the 3 *vs.* 2 mg/mL of HDM.

**FIGURE 3:**
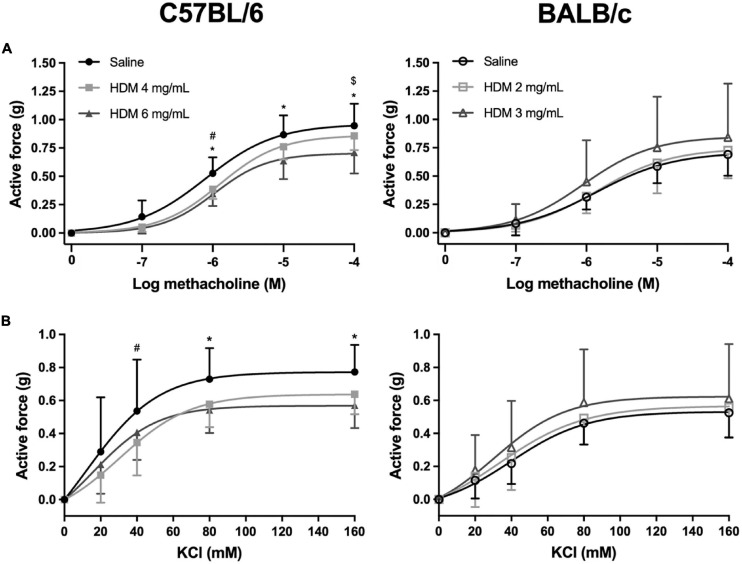
The contractile capacity of excised tracheas derived from C57BL/6 (left panels) and BALB/c (right panels) mice exposed to either saline or one of two doses of HDM. The graphs in **(A,B)** show the isometric force generated by the tracheas in response to increasing concentrations of methacholine and potassium chloride, respectively. *, #, and $ designate significant differences in C57BL/6 for saline *vs.* HDM 6 mg/mL, for saline *vs.* HDM 4 mg/mL, and for HDM 6 *vs.* 4 mg/mL, respectively (*P* < 0.05). Data are shown as means ± SD. *n* = 10 mice per group.

The isometric force generated by excised tracheas derived from C57BL/6 and BALB/c mice exposed to either saline or one of two doses of HDM in response to incremental concentrations of methacholine and KCl is depicted in Figure 3. While the repeated measures two-way ANOVA indicates that HDM exposure significantly decreased tracheal contraction in C57BL/6 mice (*p* < 0.0001), it did not influence contraction in BALB/c mice. This was true for both methacholine and KCl. *Post hoc* analyses demonstrate that *in vivo* exposure to 4 mg/mL of HDM significantly decreased the *ex vivo* contraction of tracheas in response to 10^–6^ M of methacholine and in response to 40 mM of KCl. The same tests also demonstrate that *in vivo* exposure to 6 mg/mL of HDM significantly decreased the *ex vivo* contraction of tracheas in response to 10^–6^, 10^–5^, and 10^–4^ M of methacholine and in response to 80 and 160 mM of KCl. There was no significant difference between doses of 4 *vs.* 6 mg/mL of HDM for the contraction of tracheas derived from C57BL/6 mice, except for the highest concentration of methacholine (10^–4^ M), which was significantly lower for tracheas derived from mice exposed to 6 *vs.* 4 mg/mL of HDM.

**FIGURE 4:**
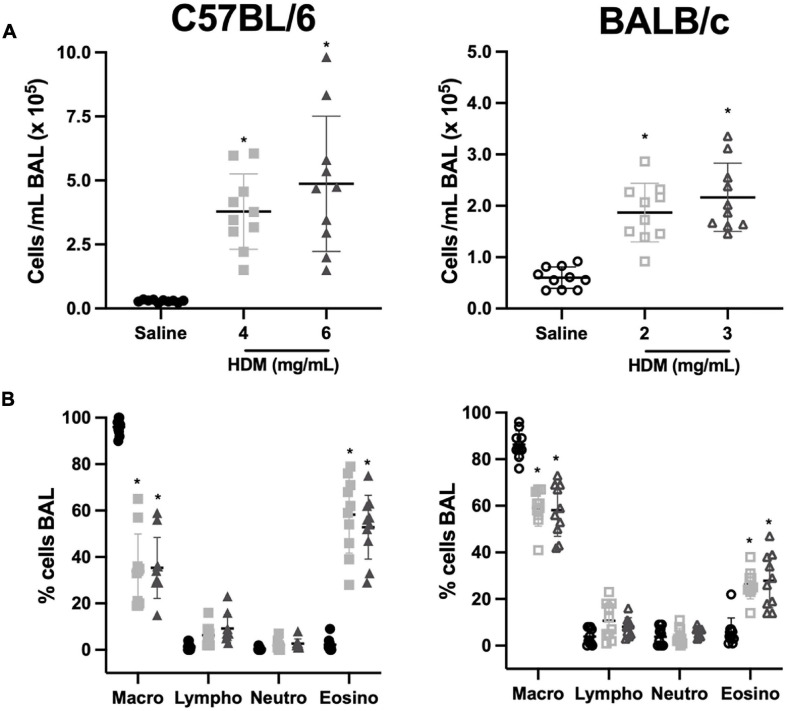
Inflammatory cells in bronchoalveolar lavages of C57BL/6 (left panels) and BALB/c (right panels) mice exposed to either saline or one of two doses of HDM. The scatter plots in **(A,B)** show the number of total cells/mL and the differential cell counts in percentages, respectively. Note that the scale on the *y*-axes in **(A)** is twice greater for C57BL/6 than BALB/c mice. *Designates significantly different from saline-treated mice within the same mouse strain (*P* < 0.05). Data are shown as means ± SD. *n* = 10 mice per group. Macro, macrophages; Lympho, lymphocytes; Neutro, neutrophils; and Eosino, eosinophils.

The number of total cells per mL of BAL in C57BL/6 and BALB/c mice exposed to either saline or one of two doses of HDM is depicted in Figure 4A. In both C57BL/6 and BALB/c mice, the number of total cells increased significantly in response to both doses of HDM. For either strain, the increases were not significantly different between the two doses of HDM. However, the increases caused by HDM were approximately twice greater in C57BL/6 than BALB/c mice. The differential cell counts in all groups are depicted in Figure 4B. In C57BL/6 mice, macrophages, lymphocytes and eosinophils were significantly increased by 3.1, 52.0, and 371.5-fold, respectively, in response to 4 mg/mL of HDM, and by 5.3, 104.8, and 402.8-fold, respectively, in response to 6 mg/mL of HDM. In BALB/c mice, only macrophages and eosinophils were significantly increased by 1.1 and 11.2-fold, respectively, in response to 2 mg/mL of HDM, and by 1.4 and 14.5-fold, respectively, in response to 3 mg/mL of HDM. For each cell type in either strain, the increases were not significantly different between the two doses of HDM. Notably, the number of eosinophils were approximately fourfold greater in C57BL/6 than BALB/c mice after HDM exposure.

**FIGURE 5:**
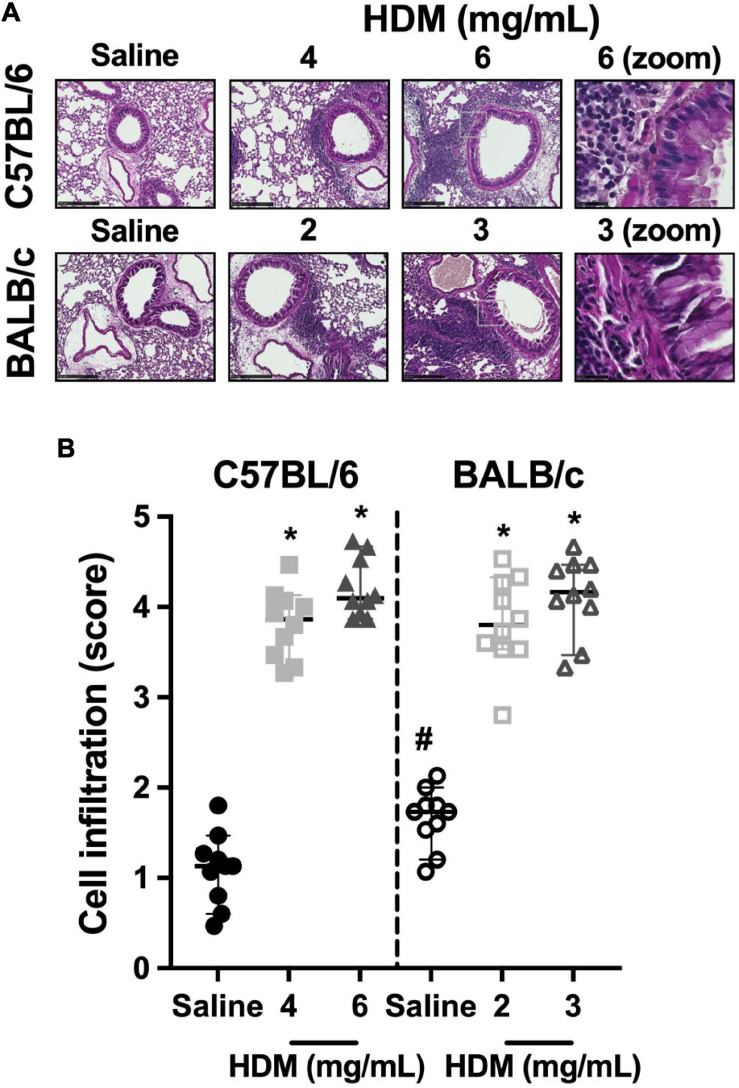
Inflammatory cell infiltrates within the lung tissue. The images in **(A)** show representative lung sections from C57BL/6 (upper panels) and BALB/c (lower panels) mice exposed to incremental doses of house dust mite (HDM) (from left to right). The zone enclosed by the gray square on images of the third row is zoomed on the next image on the right. The scale bar is 250 μm for the six first images from the left and 25 μm for the last two images on the right. An inflammatory score was assigned to each of these images and average results for each mouse in all groups are presented in the scatter plot shown in **(B)**. * and # designate significantly different from saline-treated mice within the same mouse strain and from the other mouse strain exposed to the same treatment, respectively (*P* < 0.05). Data are shown as medians with 95% confidence intervals. *n* = 10 mice per group.

The infiltration of the lung tissue with inflammatory cells in C57BL/6 and BALB/c mice exposed to either saline or one of two doses of HDM is depicted in Figure 5. In both C57BL/6 and BALB/c mice, cellular infiltration increased significantly in response to both doses of HDM. For either strain, the increases were not significantly different between the two doses of HDM. BALB/c mice demonstrated a greater infiltration of inflammatory cells compared to C57BL/6 mice after exposure to saline. However, the degree of infiltration after HDM exposure was similar between the two mouse strains.

**FIGURE 6:**
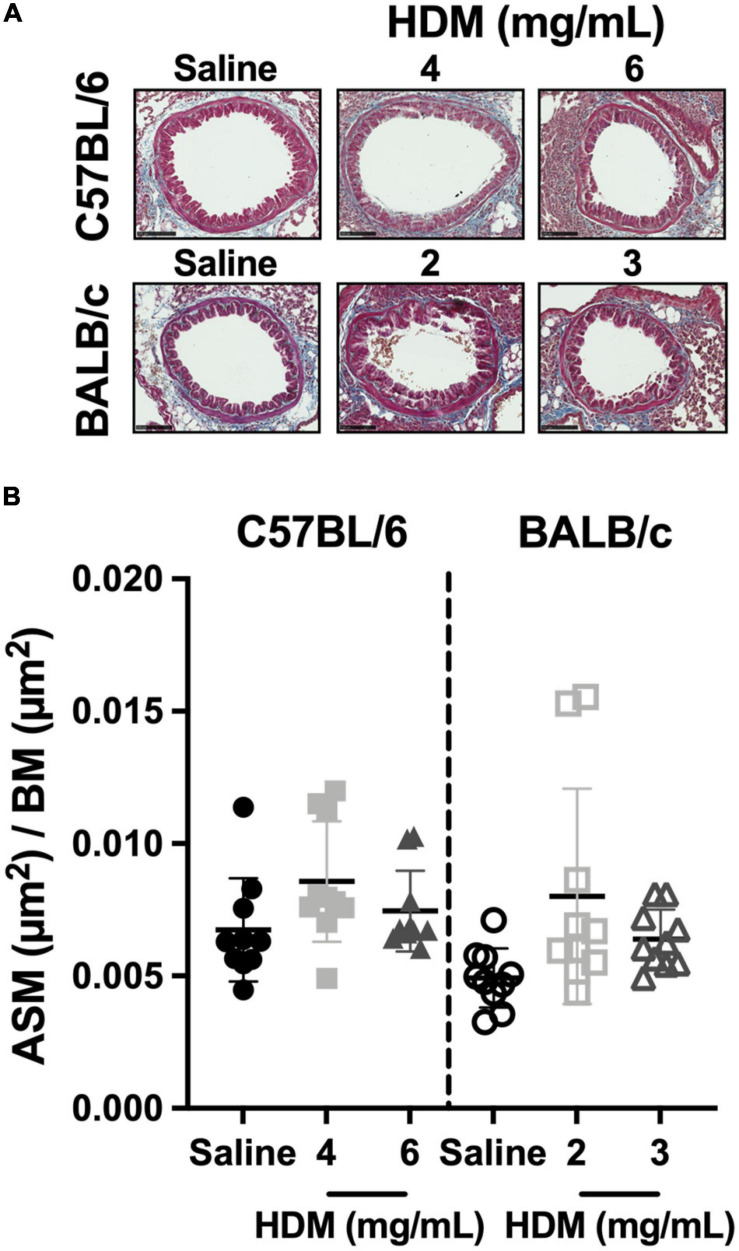
The content of smooth muscle within the airway wall. The images in **(A)** show representative lung sections from C57BL/6 (upper panels) and BALB/c (lower panels) mice exposed to incremental doses of house dust mite (HDM) (from left to right). The scale bar is 100 μm. For each bronchus analyzed, the area occupied by the airway smooth muscle (ASM) was divided by the square of the basement membrane (BM) perimeter and average results for each mouse in all groups are presented in the scatter plot shown in **(B)**. Data are shown as means ± SD. *n* = 10 mice per group.

The ASM content within the airway wall of C57BL/6 and BALB/c mice exposed to either saline or one of two doses of HDM is depicted in Figure 6. The content of ASM was neither affected by HDM nor different between the two mouse strains.

**FIGURE 7:**
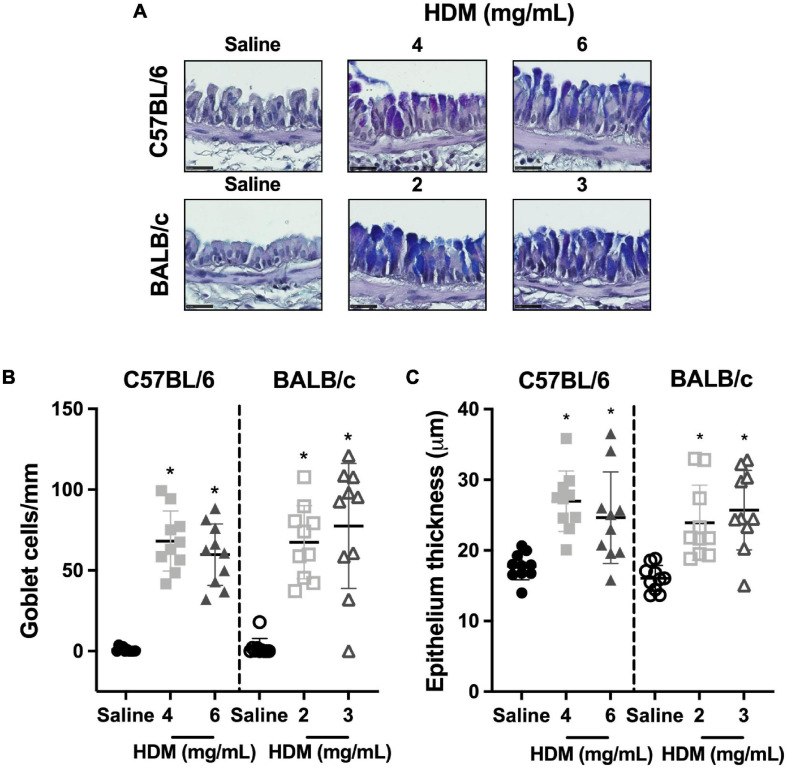
Goblet cell counts and epithelium thickness. The images in **(A)** show representative lung sections from C57BL/6 (upper panels) and BALB/c (lower panels) mice exposed to incremental doses of house dust mite (HDM) (from left to right). The scale bar is 25 μm. For each of these images, the number of goblet cells/basement membrane perimeter and the area occupied by the epithelium/basement membrane perimeter were calculated and average results for each mouse in all groups are presented in scatter plots shown in **(B,C)**, respectively. *Designates significantly different from saline-treated mice within the same mouse strain (*P* < 0.05). Data are shown as means ± SD. *n* = 10 mice per group.

The goblet cell counts and the epithelium thickness in C57BL/6 and BALB/c mice exposed to either saline or one of two doses of HDM are depicted in Figure 7. In both C57BL/6 and BALB/c mice, the goblet cell counts (Figure 7B) and the epithelium thickness (Figure 7C) increased significantly in response to both doses of HDM. For either strain, the increases were not significantly different between the two doses of HDM. The goblet cell counts and the epithelium thickness were also not significantly different between the two mouse strains.

## Discussion

The present study investigated the underlying mechanisms accounting for the different susceptibility to develop AHR upon pulmonary allergic inflammation between C57BL/6 and BALB/c mice. Although previous studies comparing these two strains in models of pulmonary allergic inflammation abound (Table 2), we are aware of only one study showing such comparisons in the model used herein (Sahu et al., 2010); i.e., intranasal exposure to HDM once daily for 10 consecutive days without prior intraperitoneal sensitization. This model is considered more genuine to human pathology (Doras et al., 2018), mainly because: (1) its route of sensitization mimics natural exposure to airborne allergens through the nasal mucosa; and (2) HDM is an allergen for which a large proportion of humans develops atopy. Another unique feature of our study is that we doubled allergen doses in the less susceptible strain in an attempt to promote AHR by furthering inflammation. Despite increasing BAL inflammation by more than twice and achieving same levels of tissue inflammation, goblet cell hyperplasia and epithelium thickness compared to BALB/c mice, C57BL/6 mice remained normoresponsive. In contradistinction with our hypothesis, the development of AHR in BALB/c mice and the lack thereof in C57BL/6 mice was not due to an increased contractility of ASM in the former and the lack thereof in the latter. It was rather caused by an acquired hypocontractility in C57BL/6 mice.

Innate airway responsiveness (i.e., in the absence of induced inflammation) is generally greater in BALB/c *vs.* C57BL/6 mice (Levitt and Mitzner, 1989; Duguet et al., 2000; Leme et al., 2010; Berndt et al., 2011). Yet, the magnitude and the direction of these different responses depend on the readout used to assess airway responsiveness. For example, when R_N_ (Newtonian resistance, which is an indicator of resistance to airflow within conducting airways) is used to assess airway responsiveness, BALB/c mice are more responsive than C57BL/6 mice (Berndt et al., 2011). However, when H (i.e., tissue elastance) is used to assess airway responsiveness, BALB/c mice are sometimes more responsive than C57BL/6 mice (Berndt et al., 2011). To add to the confusion, when Penh (i.e., enhanced pause) is used to assess airway responsiveness, C57BL/6 mice are sometimes equally (Whitehead et al., 2003) or more responsive (Zhu and Gilmour, 2009; Berndt et al., 2011; Kelada et al., 2011) than BALB/c mice. It is worth mentioning though that Penh is no longer considered suitable to assess airway responsiveness (Mitzner and Tankersley, 2003; Adler et al., 2004; Bates et al., 2004).

In the case of acquired AHR (i.e., induced by inflammation), the general consensus is again that BALB/c mice are more prone than C57BL/6 mice (Table 2). A few exceptions were reported, however, showing no significant differences between strains (Brewer et al., 1999; Kearley et al., 2009; Kodama et al., 2010) or even the opposite (Kodama et al., 2010; Chang et al., 2013). In the latter cases, where it was shown that the magnitude of the acquired AHR was greater in C57BL/6 than BALB/c mice, it was attributed to a different route of sensitization (Kodama et al., 2010) (epicutaneous instead of peritoneal, nasal or tracheal) or the use of an atypical adjuvant (Chang et al., 2013) (Freund’s adjuvant with pertussis toxin instead of aluminum hydroxide or none). The time at which the degree of airway responsiveness is assessed after the last allergenic exposure should also be taken into consideration, as the kinetics was reported to differ between the two mouse strains (Whitehead et al., 2003; Kearley et al., 2009). In particular, AHR seemed to recede more quickly in C57BL/6 mice (Kearley et al., 2009).

In the study that has used an identical model of pulmonary allergic inflammation as ours (Sahu et al., 2010), the degree of airway responsiveness was greater in BALB/c than C57BL/6 mice after exposure to HDM. Unfortunately, however, the degree of airway responsiveness was not measured in naïve (i.e., not exposed to HDM) mice, making it impossible to compare the magnitude of the acquired AHR between strains (Sahu et al., 2010). Our study demonstrated that BALB/c mice, but not C57BL/6 mice, acquire AHR in this model.

In other models of pulmonary allergic inflammation, the increased susceptibility of BALB/c mice to develop AHR compared to C57BL/6 mice was sometimes ascribed to an increased propensity to accumulate inflammatory cells within the lung tissue, either T cells (Parkinson et al., 2021), eosinophils (Takeda et al., 2001), or mast cells (Gueders et al., 2009). In the present study, we doubled the doses of HDM in C57BL/6 mice, which has led to a twofold and a fourfold greater increases in BAL total inflammatory cells and eosinophils, respectively, compared to BALB/c mice. These increased BAL cell counts in C57BL/6 mice (Figure 4A) resulted in a degree of inflammatory cell infiltration within the lung tissue that was equivalent to the one observed in BALB/c mice (Figure 5). Yet, C57BL/6 remained normoresponsive, suggesting that a different degree of infiltration of inflammatory cells within the tissue may not be the factor accounting for the different susceptibility to develop AHR upon pulmonary allergic inflammation between C57BL/6 and BALB/c mice.

Strangely, among all studies comparing the degree of airway responsiveness between C57BL/6 and BALB/c mice after exposure to offending triggers, none of them measured (and then compared between strains) the change in ASM contractility caused by inflammation (Table 2). A few other studies merit to be discussed though.

One study compared the contractility of excised tracheal segments between C57BL/6 and BALB/c mice that had been either exposed or not to pulmonary allergic inflammation (Herz et al., 1998). This later study did not measure the degree of *in vivo* airway responsiveness. The contractility of ASM was assessed by measuring the response to electrical field stimulation (EFS). More specifically, the readout to assess ASM contractility was the frequency causing 50% (ES50) of the maximal response. There was no difference between strains in mice not exposed to pulmonary allergic inflammation. However, in both strains, the ES50 decreased with pulmonary allergic inflammation, decreasing more in BALB/c than C57BL/6 mice (Herz et al., 1998). This would suggest an acquired ASM hypercontractility in both strains, being more pronounced in BALB/c mice. However, a previous study had reported similar findings in BALB/c mice but also showed that, in contrast to ES50, the response to methacholine was not affected by pulmonary allergic inflammation (Larsen et al., 1992), which is consistent with our finding. In combination, these studies suggested that the decrease in ES50 in tracheas derived from mice exposed to pulmonary allergic inflammation (Herz et al., 1998) is more relevant to the neural control of their airways than a true change in ASM contractility.

Previous comparisons of responsiveness between C57BL/6 and BALB/c mice were also performed in lung isolated perfusion system (Held and Uhlig, 2000; Landgraf and Jancar, 2008). While the lung from BALB/c mice was more responsive than the lung from C57BL/6 mice in one study (Held and Uhlig, 2000), the other study showed the opposite (Landgraf and Jancar, 2008). The increased responsiveness in the former was not restricted to methacholine, as the lung of BALB/c mice was also more responsive to endothelin-1 and the thromboxane A_2_ analog U-46619 (Held and Uhlig, 2000). Because of these discrepancies, we do not think, at least at this point, that these previous experiments can help us interpreting our findings.

It is also worthy to mention that comparisons in tracheal ASM contractility between C57BL/6 and BALB/c mice were previously performed before and after *in vitro* exposures to single inflammatory stimuli, including tumor necrosis factor α (TNFα), lipopolysaccharide (LPS) and poly-inosinic:polycytidylic acid (poly I:C) (Safholm et al., 2011). Consistent with our findings, this study demonstrated no difference in ASM contractility between C57BL/6 and BALB/c mice in untreated preparations. The response to a muscarinic agonist (carbachol) was also not affected by *in vitro* exposure to any of the tested inflammatory stimulus in both strains. However, hypercontractility to serotonin and bradykinin was acquired in both strains after exposure to inflammatory stimuli, and the magnitudes of these responses were greater in ASM from BALB/c than C57BL/6 mice. The authors concluded that these different inter-strain responses may contribute to the increased propensity of BALB/c mice to develop AHR in a context of inflammation (Safholm et al., 2011). Although this type of study is useful to assess the direct influence of single inflammatory mediators on ASM contractility, it is important to understand that exposure to a single inflammatory stimulus does not recapitulate the whole spectrum of cellular and molecular events occurring *in vivo* upon pulmonary allergic inflammation. There are plenty of inflammatory mediators that are dysregulated in a context of pulmonary allergic inflammation that can either increase or decrease the contractility of ASM (Auger et al., 2016; Gazzola et al., 2020). Therefore, although Safholm et al. (2011) study clearly demonstrated, once again, that the contractility of ASM is labile, the relevance of their findings to the inter-strain difference obtained in the present study in response to HDM is uncertain.

Another previous study reported different changes in *ex vivo* ASM contractility induced by *in vivo* exposure to an allergen, although this was not in C57BL/6 and BALB/c mice but in ASW/SnJ and SJL/J mice (Fan et al., 1997). The latter study did not measure the degree of *in vivo* airway responsiveness. It was thus not possible to determine whether the acquired ASM hypercontractility caused by pulmonary allergic inflammation in SJL/J mice, and the lack thereof in ASW/SnJ, translated into a different change in the degree of *in vivo* airway responsiveness between mouse strains. Yet, this study confirmed that *ex vivo* ASM contractility is modulated by *in vivo* pulmonary allergic inflammation (*viz.* ASM contractility is labile) and this degree of modulation is strain specific. In our study, we show that BALB/c mice became hyperresponsive while their ASM remained normocontractile and that C57BL/6 mice remained normoresponsive while their ASM became hypocontractile. It represents the second study to suggest that the different susceptibility of C57BL/6 and BALB/c mice to develop AHR upon pulmonary allergic inflammation is ascribed to a different modulation of ASM contractility. The first study was the one from Kelada et al. (2011). These authors have shown that while BALB/c mice developed AHR in response to pulmonary allergic inflammation, C57BL/6 mice inversely developed airway hyporesponsiveness. They used the purified single protein *Der p 1*, the immunodominant allergen from the *Dermatophagoides pteronyssinus* species of HDM. The protocol consisted of sensitizing the mice with two intraperitoneal injections without adjuvant followed by a single orotracheal challenge. The mice were then studied 72 h after the challenge. Their protocol was therefore different from ours. Yet, the conclusions drawn were very much the same. Although they did not measure ASM contractility directly, they demonstrated that several genes, including many G protein-coupled receptors involved in ASM contraction, were downregulated by pulmonary allergic inflammation in C57BL/6 but not BALB/c mice. Of particular interest was the downregulation of the M2 muscarinic receptor. The authors concluded that the hyporesponsiveness acquired by C57BL/6 mice in response to pulmonary allergic inflammation was probably due to a decreased contractility of ASM. We extend these findings by actually showing that ASM derived from HDM-exposed C57BL/6 mice generates less force in response to both methacholine and KCl. Taken together, these results strongly suggest that ASM from C57BL/6 mice becomes hypocontractile in response to pulmonary allergic inflammation.

How normocontractile ASM can lead to AHR in BALB/c mice and how hypocontractile ASM can lead to a normal degree of airway responsiveness in C57BL/6 mice can be baffling for some. However, these notions are very well understood (Bosse et al., 2010). Hypercontractility of ASM is not required to cause AHR (Bosse, 2021). The combined effects of pulmonary inflammation and ASM contraction are not only additive but usually synergistic in the manifestation of AHR (Bosse et al., 2010). It is thus expected that in the presence of inflammation, a normal ASM contraction should lead to AHR, as we observed in BALB/c mice. In fact, this was previously suggested in BALB/c mice (Wagers et al., 2004; Wagers et al., 2007). Based on computational modeling, Wagers et al. (2004, 2007) convincingly demonstrated that the development of AHR upon pulmonary allergic inflammation in BALB/c mice can be entirely explained by airway wall thickening and small airway closure. It is thus not totally surprising that the inter-strain difference in the susceptibility to develop AHR upon pulmonary allergic inflammation was not due to an acquired hypercontractility in BALB/c mice. The lack of AHR in C57BL/6 mice was more puzzling in that matter, since this strain clearly develops inflammation that is often worse than the one seen in BALB/c mice (Zhang et al., 1997; Morokata et al., 1999; Takeda et al., 2001; Whitehead et al., 2003; Gueders et al., 2009; Van Hove et al., 2009; Kelada et al., 2011; Evans et al., 2015). We should have anticipated a counterbalancing phenomenon, such as an acquired ASM hypocontractility, to explain their normoresponsiveness.

The present study has some limitations. First, we have used the trachea to assess the contractility of ASM. The validity of our findings thus rests on the assumption that the ASM from the trachea is appropriate to assess the overall contractility and that the ASM from other airways are similarly affected by HDM. Previous studies comparing ASM derived from the trachea *versus* lower airways have generally found no differences in contractility (Gunst and Stropp, 1988; Jiang and Stephens, 1990; Ijpma et al., 2015). However, ASM from different locations within the airway tree are sometimes, but not always (Ijpma et al., 2015), differently affected in asthma (Ijpma et al., 2020), heaves (Matusovsky et al., 2016), and murine model of asthma (Donovan et al., 2013). More precisely, while asthma (or asthma-like conditions) is sometimes associated with increased contractility of the peripheral airways but not with changes in tracheal contractility (Matusovsky et al., 2016; Ijpma et al., 2020), it is sometimes associated with increased contractility of the trachea and a decreased contractility of peripheral airways (Donovan et al., 2013). A second limitation of our study is that the underlying molecular mechanisms were not investigated. For example, previous studies have shown that IL-4 and IL-13 signaling are required for the manifestation of AHR in BALB/c mice exposed to HDM (Johnson et al., 2007; McKnight et al., 2020). Further studies will be required to determine whether these types of signaling pathways are ultimately shaping the different susceptibility of C57BL/6 and BALB/c mice to develop AHR by differently modulating ASM contractility.

## Conclusion

Our results suggest that the lack of AHR in the presence of pulmonary allergic inflammation may sometimes be ascribed to a decrease in the contractility of ASM. This is relevant to humans because not everyone with atopy or pulmonary inflammation exhibits AHR. A failure to downregulate ASM contractility under pulmonary inflammation may allow ASM to remain normocontractile and, thereby, cause AHR upon activation by synergizing with inflammation. We propose that mechanisms downregulating ASM contractility deserve further investigations as they may provide alternative and important clues for treatments.

## Data Availability Statement

The raw data supporting the conclusions of this article will be made available by the authors, without undue reservation.

## Ethics Statement

The animal study was reviewed and approved by the Committee of Animal Care of Université Laval in accordance with the guidelines of the Canadian Council on Animal Care (protocol 2018-046-2).

## Author Contributions

All the authors edited the manuscript, and read and approved the final manuscript. MB developed the experimental design, performed the laboratory experiments pertaining to respiratory mechanics, histology and the contractile assays with excised tracheas, analyzed the data, and wrote the manuscript. CH developed the experimental design, performed some of the laboratory experiments pertaining to BAL and histology, and analyzed the data. AD-M assisted in laboratory experiments pertaining to contractile assays with excised tracheas. FK performed the laboratory experiments pertaining to saline or HDM exposure. YB secured funding, developed the experimental design, analyzed the data, and wrote the manuscript.

## Conflict of Interest

The authors declare that the research was conducted in the absence of any commercial or financial relationships that could be construed as a potential conflict of interest.
